# Clarifying pathways to poor psychological health: The mediating role of psychosocial factors in the relationship between general psychopathology and quality of life impairment in patients diagnosed with schizophrenia

**DOI:** 10.1002/jclp.22747

**Published:** 2019-01-23

**Authors:** Jianlin Liu, Edimansyah Abdin, Swapna Verma, Kang Sim, Siow Ann Chong, Mythily Subramaniam

**Affiliations:** ^1^ Research Division Institute of Mental Health Singapore Singapore; ^2^ Department of Early Psychosis Intervention Institute of Mental Health Singapore Singapore; ^3^ Department of General Psychiatry Institute of Mental Health Singapore Singapore

**Keywords:** emotional distress, mediation, psychopathology, quality of life, schizophrenia

## Abstract

**Objectives:**

The present study examines the latent factor structure of general psychopathology and investigates the mediating role of unmet psychosocial concerns, motivation, and medication side effects in the relationship between general psychopathology and quality of life (QOL) impairment in patients with schizophrenia.

**Methods:**

A total of 251 patients completed self‐report measures of unmet psychosocial concerns, motivation, medication side effects, and physical/mental QOL impairment. The severity of schizophrenia was assessed on the Positive and Negative Syndrome Scale.

**Results:**

Exploratory factor analysis revealed one latent factor (emotional distress) of general psychopathology. Mediation path analyses controlling for confounding variables revealed significant indirect effects of unmet psychosocial concerns, motivation, and medication side effects on emotional distress and physical/mental QOL impairment.

**Conclusions:**

Our findings suggest that identifying optimal methods of managing co‐occurring emotional distress as well as secondary psychosocial factors on psychological health may improve QOL among patients diagnosed with schizophrenia.

## 1 INTRODUCTION

Schizophrenia is a serious, psychiatric condition that is characterized by a cluster of symptoms including positive symptoms (e.g., hallucinations, delusions), negative symptoms (e.g., blunted affect, emotional withdrawal), and general psychopathology (e.g., depression, anxiety; Upthegrove, Marwaha, & Birchwood, 2017). The global prevalence of schizophrenia has risen from 13.1 to 20.9 million in the last three decades (Charlson et al., 2018). Schizophrenia is a disabling condition as the typical onset is in early adulthood, which disrupts several important processes, such as pursuing an education, seeking employment, forming romantic relationships, and becoming independent of the family (Charlson et al., 2018). The subsequent negative symptoms and cognitive impairments further impede an individual from getting back on track. Importantly, despite optimal treatment, it is reported that approximately two‐thirds of individuals diagnosed with schizophrenia suffer from persisting symptoms (Saha, Chant, Welham, & McGrath, 2005). Therefore, schizophrenia is one of the major contributors to the global burden of disease (Charlson et al., 2018; Upthegrove, Marwaha, & Birchwood, 2017).

While the development of medications and psychological interventions have provided some relief from the symptoms of the illness, it is acknowledged that the treatment of schizophrenia goes beyond the alleviation of symptomatology (Landolt et al., 2012; Torres‐González et al., 2014). One area which has received considerable empirical and clinical support is the importance of improving quality of life (QOL) among patients with schizophrenia (de Pinho, Pereira, Chaves, & Batista, 2018; Lu et al., 2018). Broadly speaking, QOL may be defined as an individual's sense of well‐being and satisfaction with his or her life circumstances, his or her physical and mental health status, as well as his or her access to resources and opportunities (Eack & Newhill, 2007). Converging evidence from research conducted specifically on patients diagnosed with schizophrenia has shown that QOL is conceptualized as a multidimensional construct, which comprises different important areas such as generic, disease‐specific, subjective, and objective QOL (Chou, Ma, & Yang, 2014; Eack & Newhill, 2007).

Previous research has focused on examining the prevalence, correlates, and impact of QOL impairment among patients with schizophrenia (Eack & Newhill, 2007; Sim, Mahendran, Siris, Heckers, & Chong, 2004). Recent reviews have shown that psychiatric symptoms (psychotic symptoms and general psychopathology) are negatively related to QOL in patients with schizophrenia (de Pinho et al., 2018; Lu et al., 2018). Importantly, recent studies have supported previous findings (Eack & Newhill, 2007) on the strong association between general psychopathology and QOL impairment (Gardsjord et al., 2016; Wartelsteiner et al., 2016). A common criticism in the literature pertaining to this finding is the possible inflated relationship between general psychopathology and QOL impairment (Margariti, Ploumpidis, Economou, Christodoulou, & Papadimitriou, 2015; Zeng, Zhou, Lin, Zhou, & Yu, 2015). While such concerns are important, evidence from a meta‐analysis, which has pooled findings across various studies and different measures of QOL, showed that general psychopathology is moderately associated with all areas of QOL in schizophrenia (composite QOL, subjective and objective QOL, general well‐being, and health‐related QOL; Eack & Newhill, 2007). This consistent finding reflected even in nonsymptom related measures of QOL (e.g., objective QOL), might help to address concerns that the relationship between general psychopathology and health‐related QOL impairment is unduly influenced by mood (Eack & Newhill, 2007).

More importantly, the same meta‐analysis found that general psychopathology accounted for a small amount of variance (12%) in composite QOL across studies with cross‐sectional designs (Eack & Newhill, 2007). When longitudinal studies were examined, general psychopathology explained a smaller amount of variance (8%) in QOL (Eack & Newhill, 2007). This suggests that there are potential mechanisms that might better explain the relationship between general psychopathology and QOL impairment. It is important that such mechanisms be elucidated as both nonpsychotic symptomatology and associated mechanisms may be important targets for treatments aiming to improve subjective QOL for patients diagnosed with schizophrenia (De Silva, Cooper, Li, Lund, & Patel, 2013). Unfortunately, few studies have tried to elucidate the mechanisms linking the relationship between general psychopathology and subjective QOL. This is largely due to the significant heterogeneity of nonpsychotic symptoms that fall within the broad domain of general psychopathology (Eack & Newhill, 2007). While some have proposed that depression and anxiety symptoms are more related to QOL impairment (Kuo, Ma, Kuo, Huang, & Chung, 2009), the consensus in the current literature is that the contribution of specific nonpsychotic symptom domains to QOL remains unclear (de Pinho et al., 2018; Eack & Newhill, 2007).

In addition, previous research has identified some potential mechanisms, such as unmet psychosocial concerns loss of motivation and energy, and medication side effects that influence QOL among patients diagnosed with schizophrenia (Landolt et al., 2012; Torres‐González et al., 2014). However, these potential mechanisms are often either examined as factors constituting disease‐specific QOL or as individual predictors of other areas of QOL (e.g., generic QOL; Kuo et al., 2009; Zeng et al., 2015). Few studies have considered disease‐specific concerns as potential mechanisms that link the relationship between general psychopathology and general QOL impairment. Given that there is further evidence to show that generic and disease‐specific QOL are different constructs among patients with schizophrenia (Eack & Newhill, 2007; Zeng et al., 2015), new research is needed to examine the links among general psychopathology, disease‐specific concerns, and general QOL impairment in patients diagnosed with schizophrenia. This may extend current understandings of psychopathology and QOL impairment in schizophrenia and subsequently guide psychological treatments to improve QOL (De Silva et al., 2013).

This study aims to (a) clarify the importance of different components of general psychopathology on QOL impairment by elucidating the latent factor structure of general psychopathology, and (b) draw the links among general psychopathology, disease‐specific concerns, and general QOL impairment in patients diagnosed with schizophrenia. Given the exploratory nature of the present study, we were not able to identify the specific components of general psychopathology in our hypothesis. However, in line with previous findings, we hypothesized that general psychopathology would be associated with general QOL impairment and the relationship between the components of general psychopathology and general QOL impairment would be mediated by disease‐specific concerns (unmet psychosocial concerns loss of motivation and energy, and medication side effects).

## 2 METHODS

### 2.1 Participants and procedure

The participants were 251 adult outpatients, who were seeking treatment at the Institute of Mental Health, which is the only tertiary psychiatric hospital in Singapore. Participants were invited to participate in the study during their follow‐up appointments. Participants were included in the study if they (a) were diagnosed with schizophrenia by a psychiatrist based on the DSM‐IV‐TR criteria, (b) were aged between 21 and 65 years, (c) were able to understand English, and (d) were able to provide written informed consent. Participants were excluded if (a) it was their first consultation with a psychiatrist or (b) they had severe intellectual disabilities. All participants provided written informed consent prior to inclusion in the study. The present study was unable to collect information on attrition rates due to the Personal Data Protection Act, Singapore (National Healthcare Group, [Link]) and most patients were unwilling to provide basic background information upon declining participation. This study was approved by the Hospital Institutional Review Board and the Domain Specific Review Board of the National Healthcare Group of Singapore.

### 2.2 Measures

#### 2.2.1 Sociodemographic and medical variables

The following sociodemographic information was obtained through a self‐report questionnaire: Age, gender, ethnicity, marital status, education, and employment status. Participants' primary diagnosis and psychiatric comorbidities were extracted from their medical records.

#### 2.2.2 Disease‐specific concerns

The Schizophrenia Quality of Life Scale (SQLS; Wilkinson et al., 2000) was used to assess for disease‐specific concerns among patients diagnosed with schizophrenia. The SQLS is comprised of three subscales, namely, unmet psychosocial concerns loss of motivation and energy, and medication side effects. Items were rated from 0 (*never*) to 4 (*always*). Higher scores on each subscale indicated greater concerns on that specific domain. The SQLS has been validated among patients diagnosed with schizophrenia in Singapore and it is reported to have strong psychometric properties (Luo, Seng, Xie, Li, & Thumboo, 2008).

#### 2.2.3 Psychotic symptoms and general psychopathology

The severity of the symptoms of schizophrenia (positive and negative symptoms and general psychopathology) was assessed on the Positive and Negative Syndrome Scale (PANSS; Kay, Fiszbein, & Opler, 1987), which is a 30‐item semi‐structured interview. The PANSS was administered by a trained interviewer and each symptom was rated on a 7‐point Scale from 1 (*absent*) to 7 (*extreme*). Higher ratings indicated more severe levels of psychopathology. The psychometric properties of the PANSS have been examined in the local population (Jiang, Sim, & Lee, 2013).

#### 2.2.4 Physical and mental quality of life

The Short‐Form 36 Health Survey (SF‐36; J. E. Ware & Sherbourne, 1992) was used to examine the generic quality of life across eight domains: Physical functioning, role limitations due to physical problems, bodily pain, general health, vitality, social functioning, role limitations due to emotional problems, and mental health. The physical quality of life (Physical Component Summary [PCS]) and mental quality of life (Mental Component Summary [MCS]) may be obtained by working out the mean average of the physically relevant domains and the emotionally relevant domains, respectively (J. Ware & Gandek, 1998). Higher scores on the PCS and MCS indicate a better physical and mental quality of life, respectively. The SF‐36 is shown to be reliable and valid for assessing patients diagnosed with schizophrenia in Singapore (Luo et al., 2008).

### 2.3 Statistical analysis

Descriptive and preliminary analyses were performed using the Statistical Package for Social Sciences (SPSS; version 23.0; SPSS Inc., Chicago, IL). Individual *t* tests were conducted to compare the mean scores between binary variables, while correlational analysis was performed for continuous variables. Variables found to be significantly associated with physical and mental QOL were selected as potential confounders, and these were controlled for in the respective path models.

Exploratory factor analysis (EFA) and path analysis were performed in R (version 3.4.3). Assumption testing was conducted to examine the suitability of the data for EFA. First, correlations were examined among all variables to test for factorability. Moderate to strong correlations (*r* = ≥0.3) based on Cohen's criteria are required to proceed with EFA. Second, Bartlett's test of sphericity was conducted to examine if the variables were independent. A rejection of the null hypothesis would suggest that the variables are not independent, and the data is suitable for EFA. Third, the Kaiser–Meyer–Olkin (KMO) test was conducted to assess for sampling adequacy (≥0.70). We performed a parallel analysis of the overall PANSS to determine the number of factors to be extracted (Schmitt, 2011). Parallel analysis indicated five factors that exceeded the mean eigenvalue of randomly generated data across 5,000 iterations. A five‐factor PANSS model is supported in the current literature (Wallwork, Fortgang, Hashimoto, Weinberger, & Dickinson, 2012). Interpretation of factors was based on the pattern matrix with factor loadings (≥0.4), high communalities (at least 30% explained), the proportion of variance explained, and meaningful factor conceptualizations.

Based on the structural equation modeling framework, path analysis was used to examine if unmet psychosocial concerns loss of motivation and energy, and medication side effects mediated the relationship between specific general psychopathology domains, and physical/mental QOL, while controlling for confounding variables. In Model 1 (physical QOL), we controlled for age, ethnicity, education, employment status, and PANSS positive and negative scores, while we controlled for employment status, psychiatric comorbidity, and PANSS positive and negative scores in Model 2 (mental QOL). Model fit was determined based on four indices: a nonsignificant *χ* ^2^, comparative fit index (CFI) of above 0.95, Tucker–Lewis index (TLI) of above 0.95, and root mean square error of approximation (RMSEA) of below 0.05 (Kline, 2005). Bootstrapped 95% confidence intervals (CIs) based on 5,000 iterations were used to examine the significance of indirect effects from general psychopathology to QOL impairment via unmet psychosocial concerns loss of motivation and energy, medication side effects.

## 3 RESULTS

### 3.1 Descriptive and preliminary analyses

The mean age of the sample was 39.8 years (standard deviation [*SD*] = 10.24; range: 21–62 years), 56.2% were male, 58.6% were Chinese, 19.5% Malay, 16.7% Indian, and 5.2% belonged to other ethnicities; 50.6% had completed tertiary education; 54.2% were presently employed. In terms of psychiatric comorbidity, about 33.9% of the sample had at least one psychiatric comorbidity (with the majority being comorbid depression). The participants' characteristics are summarized in Table 1. Preliminary analysis was conducted to examine potential covariates of physical QOL and mental QOL. Physical QOL was observed to be associated with age (*r* = −0.14; *p* = 0.24), ethnicity (*p* = 0.001), education status (*p* = 0.002), employment status (*p* = 0.03), positive symptoms (*r* = −0.23; *p* ≤0.001), and negative symptoms (*r* = −0.15; *p* = 0.15). Mental QOL was observed to be associated with psychiatric comorbidity (*p* = 0.02), employment status (*p* = 0.001), positive symptoms (*r* = −0.42; *p* ≤0.001), and negative symptoms (*r* = −0.23; *p* ≤0.001). Thus, the variables age, ethnicity, education status, employment status, psychiatric comorbidity, and psychotic symptoms were included as potential covariates in the path analyses.

**Table 1:** Sociodemographics and clinical variables

Variables	Total (*n* = 251)
Age	39.8 ± 10.2
Gender	
Male	141 (56.2)
Female	110 (43.8)
Marital status	
Married	32 (12.7)
Divorced/single/others	219 (87.3)
Ethnicity	
Chinese	147 (58.6)
Malay	49 (19.5)
Indian	42 (16.7)
Others	13 (5.2)
Education	
Secondary and below	124 (49.4)
Tertiary and above	127 (50.6)
Employment	
Currently employed	136 (54.2)
Unemployed	115 (45.8)
Psychiatric comorbidity	
Adjustment disorder	10 (4.0)
Anxiety	11 (4.4)
Depression	27 (10.8)
Obsessive‐compulsive disorder	9 (3.6)
Substance use/dependence	13 (5.2)
Others^a^	15 (6.0)
3‐Factor model	
PANSS positive	12.0 ± 5.43
PANSS negative	10.9 ± 5.10
PANSS general psychopathology	24.8 ± 7.94
5‐Factor model	
Positive	9.57 ± 5.54
Negative	8.41 ± 4.30
Emotional distress	9.24 ± 4.31
Cognitive dysfunction	7.49 ± 3.04
Behavioral avoidance	3.95 ± 2.80
Psychosocial concerns (SQLS)	34.9 ± 22.7
Motivation (SQLS)	36.5 ± 17.5
Side effects (SQLS)	26.5 ± 19.9
Physical QOL	49.2 ± 8.14
Mental QOL	43.6 ± 11.9

*Note*. PANSS: Positive and Negative Syndrome Scale; QOL: quality of life; SQLS: Schizophrenia Quality of Life Scale.

^a^ Others include personality disorders and mild intellectual disability.

### 3.2 Exploratory factor analysis

The factorability assumption of the 30 items of the overall PANSS was supported by generally moderate to strong correlations (*r* = ≥0.3) among the items. Given the large sample size, Bartlett's test of sphericity was appropriate and significant *χ* ^2^(*df* = 435) = 3773.3; *p* ≤0.001, which suggested that the variables were not independent. The KMO value of 0.80 indicated good sampling adequacy. An exploratory factor analysis was performed using the maximum likelihood extraction method to examine the five‐factor PANSS model and to extract the nonpsychotic latent factors. Given that the factors are expected to be correlated, an Oblimin oblique rotation was used. All items had generally acceptable communalities and appeared to load onto five latent factors, which cumulatively explained a total of 47% of the variance (Table 2). We labeled each factor based on the conceptual meaningfulness of each group of items: positive symptoms, negative symptoms, emotional distress, cognitive dysfunction, and behavioral avoidance. Factor scores were computed for each factor for subsequent analysis. While the emotional distress factor contained only general psychopathology items, the remaining four factors comprised a combination of positive, negative, and general psychopathology items. In view of the study's aim to focus on nonpsychotic latent components, we only included the emotional distress factor in the subsequent path analysis.

**Table 2:** Rotated factor loadings from pattern matrix of the PANSS

Items^a^	Loadings				
	Positive	Negative	Emotional distress	Cognitive dysfunction	Behavioral avoidance
P1 (delusions)	**0.85**	−0.02	0.05	0.09	0.06
P2 (conceptual disorganization)	0.25	−0.03	−0.01	**0.56**	−0.12
P3 (hallucinatory behavior)	**0.45**	0.03	0.35	**−0.03**	0.05
P4 (excitement)	0.16	−0.13	−0.07	0.20	−0.09
P5 (grandiosity)	**0.71**	−0.08	−0.24	0.03	−0.01
P6 (suspiciousness)	**0.44**	0.03	0.22	0.01	0.32
P7 (hostility)	0.07	0.10	0.25	0.17	0.05
N1 (blunted affect)	0.08	**0.85**	−0.01	−0.13	0.08
N2 (emotional withdrawal)	0.04	**0.91**	−0.01	−0.04	−0.01
N3 (poor rapport)	−0.04	**0.76**	−0.02	0.12	−0.07
N4 (social withdrawal)	0.01	0.09	0.02	−0.01	**0.81**
N5 (abstract thinking)	−0.06	0.25	0.06	0.38	**0.07**
N6 (spontaneity)	−0.10	**0.78**	−0.08	0.12	0.01
N7 (stereotyped)	0.01	−0.07	−0.07	**0.74**	0.11
G1 (somatic concern)	0.07	−0.04	**0.41**	0.29	0.04
G2 (anxiety)	0.19	−.06	**0.55**	−0.04	0.05
G3 (guilt feelings)	0.02	−.10	**0.65**	−0.09	−0.08
G4 (tension)	0.10	0.31	0.33	0.03	−0.10
G5 (mannerisms)	0.02	0.29	−0.06	0.11	0.07
G6 (depression)	−0.03	−0.01	**0.83**	0	0.09
G7 (motor retardation)	0.01	**0.57**	0.08	−0.04	0.15
G8 (uncooperativeness)	−0.07	**0.40**	0.07	0.21	−0.07
G9 (unusual thought)	**0.93**	0.06	0.06	−0.02	−0.03
G10 (disorientation)	−0.05	0.12	0.09	**0.50**	0.02
G11 (poor attention)	0.05	−0.06	−0.06	**0.69**	0.01
G12 (lack of insight)	0.12	0.24	0.10	0.28	−0.09
G13 (disturbance of volition)	0.01	0.22	0.08	**0.40**	0.01
G14 (poor impulse control)	0.02	0.06	**0.44**	0.17	0.001
G15 (preoccupation)	0.004	0.18	0.14	**0.55**	0.06
G16 (active social avoidance)	0.002	−0.03	−0.01	0.03	**1.0**
Eigen values	2.92	3.83	2.48	2.76	2.05
% of variance	10	13	8	9	7

*Note*. Bold values indicates items with loadings (≥0.4) on each factor.

KMO: Kaiser–Meyer–Oklin score.

^a^ KMO = 0.80; Bartlett's test of sphericity = *p* ≤0.001; total variance explained by rotated solution = 47%.

In terms of associations among specific factors, there was a strong, positive correlation between emotional distress and positive symptoms (*r* = 0.54; *p* ≤0.001), but not between emotional distress and negative symptoms (*r* = 0.08; *p* = 0.21). There were moderate, positive correlations between emotional distress and cognitive dysfunction (*r* = 0.18; *p* = 0.005), and emotional distress and behavioral avoidance (*r* = 0.36; *p* ≤0.001). Positive symptoms were associated with more cognitive dysfunction (*r* = 0.34; *p* ≤0.001) and behavioral avoidance (*r* = 0.42; *p* ≤0.001). Similarly, negative symptoms were associated with more cognitive dysfunction (*r* = 0.25; *p* ≤0.001) and behavioral avoidance (*r* = 0.30; *p* ≤0.001).

### 3.3 Path analyses

Two hypothesized mediation models were constructed to examine disease‐specific concerns (unmet psychosocial concerns loss of motivation and energy, and medication side effects) as a combined potential mediator for the link between emotional distress and physical QOL (Model 1) and mental QOL (Model 2). Model 1 provided a good fit to the data, *χ* ^2^ = 677.29, *df* = 34, *p* ≤0.001, CFI = 0.99, TLI = 0.95, RMSEA = 0.06. First, emotional distress has significant direct effects on unmet psychosocial concerns *b* = 2.63, standard error (*SE*) = 0.32, *β* = 0.50, *p* ≤0.001; loss of motivation and energy, *b* = 1.35, *SE* = 0.29, *β* = 0.33, *p* <0.001; and medication side effects, *b* = 1.76, *SE* = 0.26, *β* = 0.38, *p* ≤0.001. Second, unmet psychosocial concerns *b* = 0.09, *SE* = 0.03, *β* = 0.25, *p* = 0.01; loss of motivation and energy, *b* = −0.09, *SE* = 0.04, *β* = *−*0.19, *p* = 0.01; and medication side effects, *b* = −0.24, *SE* = 0.34, *β* = *−*0.59, *p* ≤0.001, have significant direct effects on physical QOL. The direct effect of emotional distress on physical QOL was not significant, *p* = 0.53

**Figure 1:**
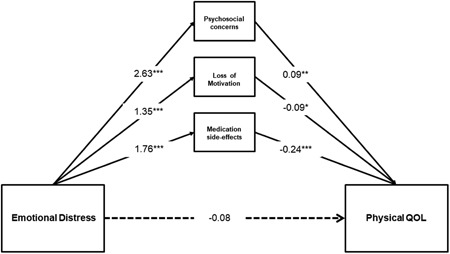
Hypothesized full mediation path model with adjustment for age, ethnicity, education, employment status, and PANSS positive and negative scores. Paths depict unstandardized *β* coefficients of direct effects of emotional distress on physical QOL, unmet psychosocial concerns loss of motivation and energy, and medication side effects; direct effect of unmet psychosocial concerns on physical QOL, direct effect of loss of motivation and energy on physical QOL, and direct effect of medication side effects on physical QOL. *p ≤0.05, **p ≤0.01, ***p ≤0.001. PANSS: Positive and Negative Syndrome Scale; QOL: quality of life

More importantly, the findings revealed that there was a significant total indirect effect of emotional distress on physical QOL via unmet psychosocial concerns loss of motivation and energy, and medication side effects, *b* = −0.31, *SE* = 0.09, 95% CI [−0.49, −0.12]. In addition, the specific indirect effects of emotional distress on physical QOL via unmet psychosocial concerns *b* = 0.24, *SE* = 0.10, 95% CI [0.07, 0.44]; loss of motivation and energy, *b* = −0.12; *SE* = 0.05; 95% CI [−0.23, −0.02]; medication side effects, *b* = −0.42, *SE* = 0.09, 95% CI [−0.60, −0.26] were significant. This suggests that unmet psychosocial concerns loss of motivation and energy, and medication side effects mediated the relationship between emotional distress and physical QOL. The path model accounted for 30% of the variance in physical QOL. Figure 1 illustrates the results of testing Model 1 via path analysis.

Similar to the first model, Model 2 provided a good fit to the data, *χ* ^2^ = 784.74, *df* = 26, *p* ≤0.001, CFI = 0.99, TLI = 0.98, RMSEA = 0.04. First, emotional distress has significant direct effects on unmet psychosocial concerns *b* = 2.65, *SE* = 0.30, *β* = 0.50, *p* ≤0.001; loss of motivation and energy, *b* = 1.38, *SE* = 0.29, *β* = 0.34, *p* ≤0.001; and medication side effects, *b* = 2.00, *SE* = 0.26, *β* = 0.43, *p* ≤0.001. Second, unmet psychosocial concerns *b* = −0.28, *SE* = 0.04, *β* = −0.53, *p* ≤0.001; and loss of motivation and energy, *b* = −0.16, *SE* = 0.04, *β* = *−*0.24, *p* ≤0.001; have significant direct effects on mental QOL. The direct effect of medication side effects on mental QOL was not significant, *b* = 0.02, *SE* = 0.04, *β* = 0.04, *p* = 0.60. The direct effect of emotional distress on mental QOL was significant, *p* = 0.02.

**Figure 2:**
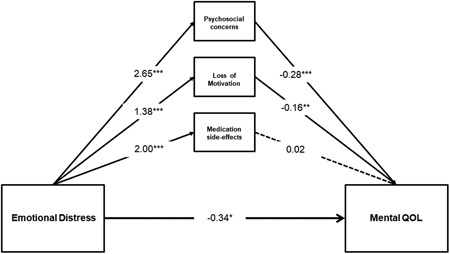
Hypothesized partial mediation path model with adjustment for employment status, psychiatric comorbidity, and PANSS positive and negative scores. Paths depict unstandardized *β* coefficients of direct effects of emotional distress on mental QOL, unmet psychosocial concerns loss of motivation and energy, and medication side effects; direct effect of unmet psychosocial concerns on mental QOL, direct effect of loss of motivation and energy on mental QOL, and direct effect of medication side effects on mental QOL. *p < 0.05, **p ≤0.01, ***p ≤0.001. PANSS: Positive and Negative Syndrome Scale; QOL: quality of life

More importantly, the findings revealed that there was a significant total indirect effect of emotional distress on mental QOL via unmet psychosocial concerns and loss of motivation and energy, *b* = −0.92, *SE* = 0.15, 95% CI [−1.22, −0.64]. In addition, the specific indirect effects of emotional distress on mental QOL via unmet psychosocial concerns *b* = −0.73, *SE* = 0.14, 95% CI [−1.03, −0.48]; and loss of motivation and energy, *b* = −0.22, *SE* = 0.10, 95% CI [−0.38, −0.09] were significant. This suggests that both unmet psychosocial concerns and loss of motivation and energy partially mediated the relationship between emotional distress and mental QOL. The path model accounted for 60% of the variance in mental QOL. Figure 2 illustrates the results of testing Model 2 via path analysis.

## 4 DISCUSSION

This present exploratory study provides preliminary evidence that adds to the existing knowledge of the association between general psychopathology and QOL impairment in patients diagnosed with schizophrenia. The identification of the latent structure of general psychopathology and the examination of specific mechanisms linking general psychopathology to QOL impairment fill an important gap in the literature, given that such knowledge was found to be lacking in previous reviews on this area. A deeper discussion of our findings will reveal important implications for further research and clinical practice.

First, the present study has identified one nonpsychotic latent factor of general psychopathology through exploratory factor analysis. Further analysis has revealed that this factor may be meaningfully labeled as emotional distress. Our findings corroborate well with previous studies that have identified a “depression” factor within a five‐factor PANSS model (Jiang et al., 2013; Wallwork et al., 2012), as well as previous reports on the high prevalence of depression among patients with schizophrenia (Charlson et al., 2018). The present study observed that emotional distress was significantly associated with both physical and mental QOL impairment. Hence, our findings address an important research gap by identifying a specific nonpsychotic, general psychopathology domain that is associated with QOL impairment in patients with schizophrenia. The present study further revealed that emotional distress was most strongly associated with more severe positive symptoms. Our finding is supported by previous research, which has reported that patients with schizophrenia and comorbid depression have more severe positive symptoms, a longer duration of illness, and poorer clinical outcomes (Gregory, Mallikarjun, & Upthegrove, 2017). Cognitive dysfunction and behavioral avoidance were observed to be moderately to strongly correlated with more positive symptoms, negative symptoms, and emotional distress. This may highlight the transdiagnostic role of cognitive dysfunction and behavioral avoidance in both schizophrenia and comorbid depression. Further research is needed to clarify the contribution of these two symptom domains to psychotic and nonpsychotic psychopathology in schizophrenia.

Second, the present study has identified specific mechanisms linking the relationship between emotional distress and QOL impairment. Specifically, our results show that disease‐specific concerns (unmet psychosocial concerns loss of motivation and energy, and medication side effects) mediated the relationship between emotional distress and physical QOL impairment. Unmet psychosocial concerns and loss of motivation and energy, but not medication side effects, mediated the relationship between emotional distress and mental QOL impairment. Previous studies have shown empirical support for each of these mechanisms and their influence on QOL impairment in patients diagnosed with schizophrenia (Landolt et al., 2012; Torres‐González et al., 2014). However, the present study extends previous knowledge by examining the role of these mechanisms in the relationship between emotional distress and QOL impairment. Importantly, our findings have clinical implications by emphasizing the need to understand and target the underlying factors of psychopathology. The sole focus on alleviating emotional distress might not be effective in the long term if disease‐specific concerns are not adequately addressed. This is due to disease‐specific concerns acting as persistent stressors that might become chronic over time and negatively affect QOL. For example, a study conducted by Staring, Mulder, Duivenvoorden, De Haan, & Van der Gaag (2009) has shown that while greater compliance to antipsychotic medications may reduce the severity of psychotic symptoms, this may also lead to adverse medication side effects, which was related to lower QOL. In the same vein, further research should explore and examine additional cognitive and effective risk factors of worry, rumination, and catastrophic thinking that might underly these disease‐specific concerns, and their link between emotional distress and QOL impairment. These transdiagnostic risk factors are often studied as cognitive emotion regulation strategies in schizophrenia (O'driscoll, Laing, & Mason, 2014). Therefore, the present study provides some preliminary support for additional research in this area.

Although the present study has revealed important implications for further research and clinical practice, they must be considered together with some limitations. First, we identified emotional distress based on a factor‐analytic approach. We do not consider this factor identical to clinical depression, and thus, we have refrained from labeling our factor as such to avoid confusion. Structured clinical interviews and standardized tests are needed to further assess for clinical depression. Hence, our hypothesized model may be considered preliminary, and further research is needed to verify its replicability. Second, some of the items in our measures may overlap with each other, and this could potentially lead to an overestimation of the hypothesized model (Eack & Newhill, 2007; Zeng et al., 2015). While this is a genuine concern, research evidence has shown that generic and disease‐specific QOL are different constructs with different sets of predictors (Eack & Newhill, 2007; Wilkinson et al., 2000; Zeng et al., 2015). In addition, from a conceptual perspective, psychopathology and QOL are also different constructs, as evident by the importance of treating symptoms and improving QOL in the literature (Landolt et al., 2012; Staring et al., 2009). Efforts have also been made in the present study to control for PANSS positive and negative symptoms that may inflate the relationship between emotional distress and QOL impairment. Given that the present study has provided preliminary evidence on the role of disease‐specific concerns, future research should investigate our hypothesized model by using more comprehensive measures that assess psychosocial concerns, motivation, and medication side effects in patients diagnosed with schizophrenia (Landolt et al., 2012; Staring et al., 2009; Torres‐González et al., 2014). Finally, the present study adopts a cross‐sectional design, and we were not able to draw firm conclusions about the directions of causality. While is possible that patients with poorer QOL may experience more severe emotional distress, these alternative models showed poorer fit to our data; physical QOL to emotional distress: *χ* ^2^ (*df*) = 742.98 (34), *p* =<0.001, CFI = 0.95, TLI = 0.76, RMSEA = 0.14, and mental QOL to emotional distress: *χ* ^2^ (*df*) = 824.62 (26), *p* ≤0.001, CFI = 0.96, TLI = 0.74, RMSEA = 0.18. Longitudinal studies are needed to verify our hypothesized model and identify causal relationships between variables. However, to our knowledge, there are no studies that have examined our hypothesized model, and thus, our findings are considered preliminary.

## 5 CONCLUSION

Notwithstanding the aforementioned limitations, the present study has identified emotional distress as a specific nonpsychotic, general psychopathology domain that is associated with QOL impairment, as well as disease‐specific concerns that mediate the relationship between emotional distress and QOL impairment. Future prospective research is needed to further examine the role of disease‐specific concerns and associated mechanisms (e.g., emotion regulation) in the relationship between emotional distress and QOL impairment in patients diagnosed with schizophrenia. More research that continues to elucidate the psychological mechanisms linking psychopathology and QOL in schizophrenia is needed to better treat and manage this multidimensional illness.
